# Insights into the Molecular Structure and Spectroscopic Properties of HONCO: An Accurate *Ab Initio* Study

**DOI:** 10.1021/acs.jpca.3c05741

**Published:** 2023-11-03

**Authors:** Cristina Puzzarini, Roberto Linguerri, Majdi Hochlaf

**Affiliations:** † Dipartimento di Chimica “Giacomo Ciamician″, Università di Bologna, Via Selmi 2, 40126 Bologna, Italy; ‡ Université Gustave Eiffel, COSYS/IMSE, 5 Bd Descartes, 77454 Champs sur Marne, France

## Abstract

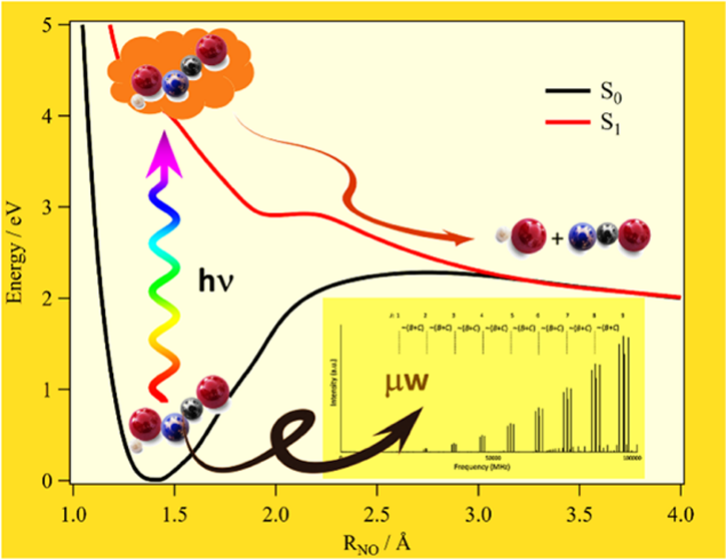

In an effort to provide the first accurate structural and spectroscopic characterization of the quasi-linear chain HONCO in its electronic ground state, state-of-the-art computational approaches mainly based on coupled-cluster (CC) theory have been employed. Equilibrium geometries have been calculated by means of a composite scheme based on CC calculations that incorporates up to the quadruple excitations and accounts for the extrapolation to the complete basis set limit and core correlation effects. This approach is proven to provide molecular structures with an accuracy better than 0.001 Å and 0.05° for bond lengths and angles, respectively. Incorporation of vibrational effects permits this level of theory to predict rotational constants with an estimated accuracy of 0.1% or better. Vibrational fundamental bands have been evaluated by means of a hybrid scheme based on harmonic frequencies computed using the CC singles, doubles, and a perturbative treatment of the triples method (CCSD(T)) in conjunction with a quadruple-ζ basis set, with all electrons being correlated, and anharmonic corrections from CCSD(T) calculations using a triple-ζ basis set, within the frozen-core approximation. Such a hybrid approach allowed us to obtain fundamental frequencies with a mean absolute error of about 1%. To complete the spectroscopic characterization, vertical electronic excitation energies have been calculated for the lowest singlet and triplet states using the internally contracted multireference configuration interaction (MRCI) method. Computations show that HONCO dissociates into OH + NCO upon the absorption of UV–vis light. In conclusion, we are confident that the highly accurate spectroscopic data provided herein can be useful for guiding future experimental investigations and supporting the characterization of this molecule in atmospheric and astrophysical media, as well as in combustion.

## Introduction

The HONCO molecule and its isomers are important species that are involved in numerous chemical processes. For instance, they are implicated in combustion of organic compounds and atmospheric reactions.^1,2^ Indeed, the stable and metastable HONCO isomeric forms have been the subject of theoretical investigations in order to elucidate their role as intermediates in the mechanisms of the CH + NO_2_, NH + CO_2_, N + HOCO and HCO + NO reactions.^3−7^ In particular, in their theoretical studies of the CH + NO_2_ reaction, Tao et al.^3,4^ characterized the HONCO isomers on the lowest singlet and triplet potential energy surfaces (PESs), at the B3LYP/6-311G(d,p) level. The relative energies of these isomers were then established by accurate CCSD(T)/6-311G(d,p) single-point computations on top of B3LYP/6-311G(d,p) optimized geometries, also incorporating zero-point-energy (ZPE) corrections. On the lowest singlet B3LYP/6-311G(d,p) PES, these authors identified 25 isomers and 50 transition states, also including some weakly bound complexes. Among these systems, the HO−N=C=O molecular chain was found to be the lowest in energy. In a previous theoretical investigation, Kulkarni and Koga,^7^ who studied this molecule in connection with the NO + XCO (X = H, F, Cl) reactions in the gas phase, concluded that HONCO, CH(=O)NO, and HNOCO, in order of increasing energy, correspond to the lowest-energy isomers at the same level of theory as above (i.e., CCSD(T)/6-311G(d,p) energy on top of B3LYP/6-311G(d,p) geometries).

The principal focus of the above-cited theoretical works on HONCO isomers was to establish their role as key intermediates in reactions that are relevant for combustion and atmospheric processes, and a very limited theoretical investigation was instead devoted to the accurate characterization of their spectroscopic properties. Differently, a more recent and thorough *ab initio* study by Tourchi et al.^8^ of the stable forms of the [H,C,N,O,O] pentatomic molecular system focused on their structural and vibrational spectroscopy characterization.^8^ Twenty isomers of HONCO lying within the 0.0–5.7 eV energy window were identified, and confirmed the previous theoretical predictions giving the chain-like form as the most stable.^8^ Tourchi et al.^8^ performed structural optimizations at the CCSD(T) level in conjunction with the aug-cc-pV*n*Z (*n* = D,T) basis sets with the extrapolation to complete basis set (CBS) limit, followed by accurate predictions of the fundamental bands. For the latter, a hybrid scheme combining CCSD(T) harmonic frequencies with MP2 anharmonic corrections was employed.

The [H,C,N,O,O] family might have some relevance in astrochemistry, and, in particular, its most stable isomer HONCO is a potential interstellar species. Indeed, isocyanates (X−NCO) can be considered prebiotic systems because they play a role in the synthesis of amino acids, in the polymerization of peptides, and in the production of nucleotides and nucleosides (see ref (9) and references therein). In the interstellar medium (ISM), in addition to the isocyanic acid (HNCO), which was among the very early molecules detected in space,^10−12^ more complex systems containing the NCO moiety, such as methyl isocyanate^13−17^ and ethyl isocyanate,^9^ have already been discovered. The observation of another element of the family would provide an important step forward because little is known about the chemistry of isocyanates in space. The presence of the isocyanate radical (NCO)^18^ and N-protonated isocyanic acid (H_2_NCO^+^)^18,19^ in the ISM suggests the possibility of incorporating the NCO moiety in molecules of increasing complexity.

So far, experimental spectroscopic data about HONCO and its related species have been very scarce. The vibrational spectra of a molecule corresponding to this molecular formula were measured by Milligan et al.,^20^ who studied the photolysis of HN_3_ in a CO_2_ cold matrix. In order to explain a set of IR absorptions recorded, the authors postulated the formation of a 1:1 compound between NH and CO_2_. Among the possible structures they proposed, the title molecule was also considered as the result of a rearrangement of the primary NH:CO_2_ compound. To further extend the analysis by Milligan et al.,^20^ accurate predictions for the IR spectrum of the possible isomers are required.

To open the way toward an experimental spectroscopic characterization of the quasi-linear chain HONCO in its electronic ground state, i.e., the most stable [H,C,N,O,O] isomer, in this work, we employed advanced electronic structure approaches based on the coupled-cluster (CC) technique. Indeed, for the assignment of the high-resolution rotational or infrared (IR) spectra, it is essential to determine molecular geometries and vibrational frequencies at the highest possible level of accuracy.^21^ To accomplish this demanding task, it is necessary to adopt state-of-the-art *ab initio* methodologies that go well beyond the standard electronic structure methods and the simple rigid rotor–harmonic oscillator approximation.^21−24^ In addition, an investigation on the low-lying electronic states of HONCO, i.e., those comprised between 0.0 and 7.0 eV above the electronic ground-state energy, is performed to elucidate the main characteristics of its UV–vis spectrum. This is achieved via an accurate multireference approach, which is capable of conveying precise information on the manifold of the lowest singlet and triplet excited electronic states.

## Computational Details

### Electronic Ground State

Quantum-chemical calculations for the electronic ground state of HONCO were carried out with the CFOUR suite of quantum chemistry programs.^25^ Most of these computations employ the CC method with singles, doubles, and a perturbative treatment of connected triple excitations^26^ (CCSD(T)). All calculations were performed in conjunction with the cc-pV*n*Z (*n* = Q-6) atomic basis sets of Dunning and co-workers.^27,28^

Best-estimated structural parameters have been evaluated by combining the gradient-scheme^29,30^ and the geometry scheme^31,32^ approaches. The former, implemented in a black-box manner in the CFOUR program, has been exploited to perform geometry optimization at the CCSD(T)/CBS+CV level, where CV denotes the incorporation of core-valence corrections. HF-SCF and CCSD(T) correlation energies are extrapolated separately using Feller’s exponential^33^ (*n* = Q-6) and *n*^–3^ (*n* = Q,5) extrapolation formula,^34^ respectively. For CCSD(T), the frozen-core (fc) approximation is employed. The CV correction is evaluated by adding to the energy gradient the contribution corresponding to the difference of all-electron (ae) and fc CCSD(T) energies, obtained with the cc-pCVQZ basis set.^35^ Overall, the CCSD(T)/CBS+CV energy gradient is defined as
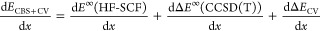
1

The geometry scheme has been employed to incorporate the contributions of the full treatment of triple excitations (fT) and the perturbative treatment of quadruple excitations (pQ) in the cluster expansion, thus leading to the CCSD(T)/CBS+CV+fT+pQ structure:
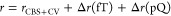
2where *r* denotes a generic structural parameter and *r*_CBS+CV_ is the corresponding value obtained from the CBS+CV geometry optimization defined by eq 1. The Δ*r*(fT) correction is evaluated as the difference between *r* values computed at the fc-CCSDT/cc-pVTZ and fc-CCSD(T)/cc-pVTZ levels, CCSDT standing for the CC singles, doubles, and triples method.^36^ The Δ*r*(pQ) contribution is given by the difference between the *r* values computed at the fc-CCSDT(Q)/cc-pVDZ and fc-CCSDT/cc-pVDZ levels, with CCSDT(Q) being the CCSDT method augmented by a perturbative treatment of quadruple excitations.^37^

The spectroscopic parameters required for predicting the rotational spectrum are the rotational and centrifugal distortion constants. In some cases, hyperfine constants (*vide infra*) might also be needed. According to vibrational perturbation theory to second order (VPT2),^38^ rotational constants consist of two terms: the equilibrium contribution, which is the dominant one (by accounting for about—usually more than—99%)^39^ and the vibrational correction, which incorporates the effects of molecular vibration. For a generic vibrational state *v*, the expression of the corresponding rotational constant *B*_*v*_^*i*^ is given by
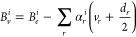
3where *B*_*e*_^*i*^ is the equilibrium rotational constant relative to the *i*-th inertial axis (*i* = *a, b*, or *c* so that, e.g., *B*^*a*^ = *A*), and only depends on the equilibrium structure (and isotopic mass composition). The second term is the vibrational correction, which is obtained by summing, over the vibrational modes *r* (v_*r*_ being the associated vibrational quantum number and *d*_*r*_ its degeneracy), the vibration–rotation interaction constants (α_*r*_^*i*^). If the vibrational ground state is considered (*B*_0_^*i*^), the second term of eq 3 becomes the half-sum of the vibration–rotation constants, and the vibrational correction is simply denoted as Δ*B*_vib_^*i*^, which is—contrary to the single α_*r*_^*i*^—devoid of any resonance.^40^ The calculation of the vibration–rotation interaction constants requires anharmonic force field computations, which were performed here at the fc-CCSD(T)/cc-pVTZ level. Additionally, the harmonic force field has been computed at the ae-CCSD(T)/cc-pCVQZ level of theory, which allows us to derive accurate quartic centrifugal distortion constants.

For predicting the rotational spectrum, in addition to the rotational and centrifugal distortion constants, the components of the electric dipole moments along the inertial axes are required in order to obtain information on the type and intensity of transitions that can be observed.^40,41^ Since for an accurate estimate of this property, incorporation of the diffuse function is important, its evaluation has been performed at the fc-CCSD(T)/aug-cc-pVTZ^27,42^ level of theory. Since HONCO is not a commercial molecule, very likely it needs to be produced on-the-fly while performing the measurements, thereby exploiting *ad hoc* techniques, such as the flash vacuum pyrolysis,^43,44^ which, however, can lead to the concomitant formation of other (unwanted) species. Therefore, characteristic features in the rotational spectrum can be very useful for the identification of a molecule of interest. These can be provided by electric and/or magnetic interactions, which occur whenever a molecule contains one or more atoms with nonzero nuclear spin *I*. These interactions split the rotational energy levels and, consequently, rotational transitions, giving rise to the so-called hyperfine structure of the rotational spectrum.^40,41^ The interaction of interest (which is the strongest one in closed-shell species) is the nuclear quadrupole coupling that takes place whenever the molecule has a quadrupolar nucleus (*I* ≥ 1) because its quadrupole moment interacts with the electric field gradient at the nucleus itself. In HONCO, the quadrupolar nucleus is the nitrogen atom. From a computational point of view, the prediction of nuclear quadrupole coupling constants, χ_*ij*_, requires the calculation of the electric field gradient at the quadrupolar nucleus
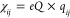
4where *i* and *j* refer to the inertial axes, *eQ* is the nuclear quadrupole moment, and *q*_*ij*_ represents the *ij*-th element of the electric field gradient tensor.^40,41^ Based on the literature on this topic (see, e.g., refs (40,45−47)), the latter has been computed at the ae-CCSD(T)/cc-pCVQZ level in order to obtain quantitative predictions.

The ae-CCSD(T)/cc-pCVQZ harmonic force field computation, in addition to the quartic centrifugal distortion constants, also provides the harmonic frequencies (denoted as ω(ae-CC/CQZ)). The anharmonic force field computation carried out at the fc-CCSD(T)/cc-pVTZ level, with the cubic and semidiagonal quartic force constants being obtained by numerical differentiation of the analytic harmonic force constants^48^ (as implemented in CFOUR), allows—within the VPT2—the derivation of vibrational frequencies (denoted as ν) beyond the harmonic approximation. To improve the prediction of fundamental frequencies, a hybrid model is considered. This approach assumes that the differences between vibrational frequencies computed at two different levels of theory are only due to the harmonic terms, and it is well tested in the literature (see, for instance, refs (49−52)). In more detail, the harmonic frequencies ω are *a posteriori* corrected by anharmonic contributions (Δν(fcCC/VTZ) = ν–ω) derived from VPT2 applied to the fc-CCSD(T)/cc-pVTZ anharmonic force field:
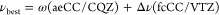
5

### Electronic Excited States

Vertical excitation energies were calculated using the internally contracted multireference configuration interaction (MRCI) method,^53,54^ where the reference wave functions were built from molecular orbital sets obtained through state-averaged complete active space self-consistent field (SA-CASSCF) computations.^55,56^ Singlet and triplet states were calculated separately. The SA-CASSCF procedures were carried out by assigning equal weights to all of the considered states, i.e., the three lowest singlet/triplet states. The molecular symmetry properties were explicitly used, with all calculations performed in the *C*_*s*_ symmetry group. The SA-CASSCF active space consists of 14 electrons in 13 active molecular orbitals, resulting in around 37 × 10^3^ and 64 × 10^3^ configuration state functions (CSFs) for the singlet and triplet states of a given symmetry species, respectively. All of these computations were performed using the aug-cc-pVQZ basis set. In the MRCI procedure, the reference wave functions were built from the CAS vectors by selecting only those configurations with CI coefficients larger than 0.01. This resulted in a number of uncontracted CSFs of around 3.9 × 10^9^ (5.9 × 10^9^) and 9.4 × 10^9^ (11.7 × 10^9^) for the singlet and triplet states of A′ (A″) symmetry, respectively. CASSCF and MRCI calculations were performed using the MOLPRO 2015.1 suite of *ab initio* programs.^57^

Since the S_0_ and T_1_ states have a monoconfigurational character, the S_0_-T_1_ transition energy has been estimated using the CCSD(T) method, which provides more accurate results than MRCI. For the purpose, we employed the fc-CCSD(T)/cc-pVQZ level of theory to compute this energy difference, and we used this value, along with the MRCI energies for the upper triplet states, to determine the positions (vertical energy) of the triplet excited states relative to the S_0_ state.

## Results

### Equilibrium Geometry and Rotational Spectrum

**Figure 1:**
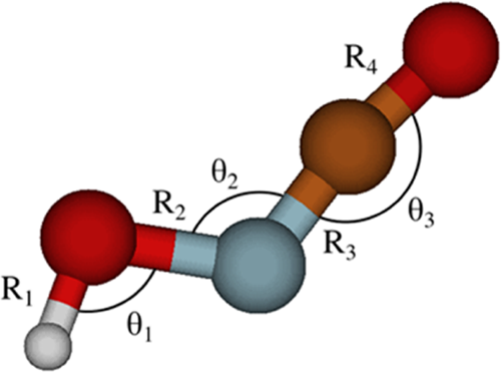
Definition of the internal coordinates of the HONCO molecule.

The HONCO molecule in its *X̃*^1^*A*′ state is a planar (*C*_*s*_), quasi-linear chain, showing a *trans*-like H−O−N−C moiety (see Figure 1). In previous theoretical investigations, this isomer of the HNCO_2_ family, in its singlet spin state, has already been identified as the lowest energy one for the [H,O,N,C,O] system (see, for example, refs (3,7,8)). The CCSD(T)/CBS+CV+fT+pQ equilibrium geometrical parameters, together with the corresponding equilibrium rotational constants, are collected in Table 1, where the comparison with the CCSD(T)/CBS results from ref (8) are also reported. Despite the fact that in ref (8) the extrapolation to the CBS limit was performed with small basis sets (aug-cc-pVDZ and aug-cc-pVTZ), the agreement between the two structures is rather good. The mean absolute difference for bond lengths is about 0.007 Å, with the present values being systematically shorter than those of ref (8). On a general basis, bond lengths shorten because of the extrapolation to the CBS limit and incorporation of the core correlation effects. In the CCSD(T)/CBS results of ref (8), the lack of the CV correction might be partially compensated by the overestimation of the extrapolation to the CBS limit due to the use of small basis sets. In particular, the CV correction shortens the bond distances by about 0.001–0.003 Å, where the smallest value applies to the OH distance (R_1_; see Figure 1), while it is nearly negligible for angles. The fT contribution is very small, this being negative (thus further reducing) for bond lengths but smaller—on average—than 0.0005 Å, and positive—on the order of +0.03°—for angles. The pQ correction goes in the opposite direction and is larger: on average, +0.001 Å and −0.1°, for distances and angles, respectively. The last comment concerns the comparison of the equilibrium rotational constants. From Table 1, it is noted that even if the two structures are in good agreement, small deviations in geometrical parameters lead to large differences in the corresponding rotational constants. These are about 1700 MHz for *A*_e_ (∼2.4%) and around 50 MHz for both *B*_e_ and *C*_e_ (∼1%).

**Table 1:** CCSD(T)/CBS+CV+fT+pQ Equilibrium Structural Parameters (for Their Definition, See Figure 1) and Rotational Constants of HONCO (*X̃*^1^*A*′)^a^

parameter	CCSD(T)/CBS + CV + fT + pQ	CCSD(T)/CBS from ref (8)
*R*_1_	0.9605	0.963
*R*_2_	1.4091	1.416
*R*_3_	1.2300	1.241
*R*_4_	1.1645	1.173
θ_1_	101.43	101.0
θ_2_	117.84	117.5
θ_3_	170.41	170.5
*A*_e_	73802.68	72100.39
*B*_e_	4850.84	4797.48
*C*_e_	4551.67	4498.17

a Bond Lengths (*R*_i_) are in Å, angles (θ_i_) are in degrees, and equilibrium rotational constants (*A*_e_, *B*_e_, and *C*_e_) are in MHz.

**Figure 2:**
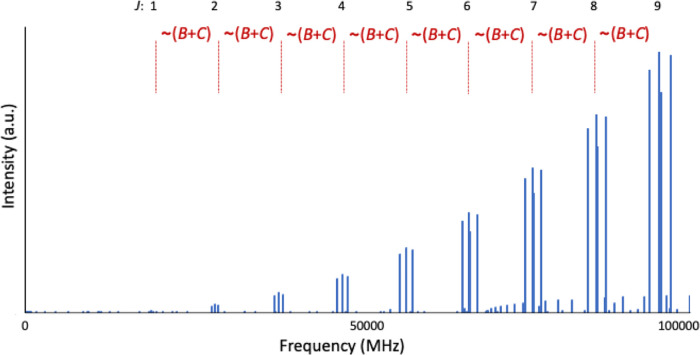
Simulation of a portion of the rotational spectrum (0–100 GHz) at the temperature of 100 K. The most intense features are ^*a*^*R*_0,1_ transitions (*J*+1_*K*_*a*_,*K*_*c*_+1_ ← *J*_*K*_a_,*K*_c__), with the *J* value of the starting energy level being reported on top of the figure.

The spectroscopic and molecular parameters of relevance to rotational spectroscopy are collected in Table 2. HONCO is a nearly prolate asymmetric rotor, with *a* and *b* being the inertial axes defining the molecular plane and *c* being perpendicular to it. The approach employed for the determination of the rotational constants (i.e., equilibrium values at the CCSD(T)/CBS+CV+fT+pQ augmented by fc-CCSD(T)/cc-pVTZ vibrational corrections) should provide predictions with a relative accuracy of better than 0.1%,^40,58^ while for quartic centrifugal distortion constants at the ae-CCSD(T)/cc-pCVQZ level, deviations from experiment are expected to be on the order of 1%.^21,59^ While the accuracy of the computational approach employed has been well tested in the literature, the molecule under consideration is a challenging case because of the quasi-linearity of the NCO moiety. Using the test γ_0_ parameter introduced in ref (60)
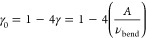
6where *A* is the rotational constant and ν_bend_ is the frequency associated with the NCO bending mode (ν_6_), our system results to lie within the well-behaved bent molecule range. In fact, the latter goes from γ_0_ = 1 to γ_0_ = 0.96, and for HONCO, a value of 0.985 is obtained. Furthermore, only rotational constant *A* is actually affected, and this has a limited impact on the prediction of the rotational spectrum. Indeed, based on the values of the electric dipole moment components reported in Table 2, the rotational spectrum of HONCO is expected to be characterized by strong *a*-type and weak *b*-type transitions, the intensity of rotational transitions being proportional to the square of the corresponding μ component. Therefore, the rotational spectrum of HONCO is dominated by strong ^*a*^*R*_0,1_ transitions^a^, which are nearly separated by the (*B* + *C*) quantity, as shown in Figure 2. The *a*-type rotational transitions (|μ_a_| = 1.75 D) are thus predicted with uncertainties that mainly reflect the accuracy of the computed rotational constants *B* and *C*, which are barely affected by the quasi-linearity issue. Therefore, an accuracy of about 0.1% is also expected for rotational transitions, especially for those little suffering from centrifugal distortion (low values of *J*). The resulting simulation of the rotational spectrum in the 0–100 GHz frequency range at 100 K is shown in Figure 2. The spectrum is clearly dominated by *a*-type transitions (^*a*^*R*_0,1_ transitions from *J* = 1 to *J* = 9 are visible), with the different *K*_*a*_-components being evident. Instead, the *b*-type transitions are barely observable because of the small value of the corresponding component of the dipole moment (|μ_b_| = 0.47 D). The ground-state rotational spectrum might, however, be affected by perturbations. This is a common feature of quasi-linear molecules and is due to a breakdown of the Watson-type asymmetric rotor Hamiltonian caused by accidental rotational level degeneracies. In fact, it might happen that *K*_*a+*1_ levels of the ground state become close in energy with the *K*_*a*_ levels of a low-lying, totally symmetric, vibrationally excited state (the v_7_ = 1 state in the present case). In such a case, the rotational spectrum can be modeled only for low values of *K*_*a*_, but an accurate line catalog can be usually still obtained.^43,61^ This effect was first described by Yamada,^62^ with the theory subsequently detailed in ref (63). For clarity purposes, the spectral simulation of Figure 2 does not incorporate the hyperfine structure due to the nitrogen quadrupole coupling constants. However, as already noted, the corresponding features might provide crucial support for the assignment of the experimental spectrum. To this aim, it is noted that the parameters reported in Table 2 are expected to have an accuracy of 0.1%, which can be further improved—if required—by incorporating vibrational corrections, which are anyway usually very small.^40,64^

**Table 2:** Computed Rotational^a^ and Quartic Centrifugal Distortion^b^ Constants;^c^ Nitrogen Quadrupole Coupling Constants^d^ and Dipole Moment Components^e^ Are Also Reported

parameter	units	value
*A*_0_	MHz	72633.43 [1169.25]
*B*_0_	MHz	4847.79 [3.05]
*C*_0_	MHz	4539.35 [12.32]
10^3^ Δ_*J*_	MHz	2.25
Δ_*K*_	MHz	17.22
Δ_*JK*_	MHz	–0.274
10^4^ δ_*J*_	MHz	4.35
10^2^ δ_*K*_	MHz	2.22
χ_aa_	MHz	4.01
χ_bb_	MHz	–1.09
χ_ab_	MHz	2.61
μ_a_	D	–1.75
μ_b_	D	0.47

a *A*_0_, *B*_0_, *C*_0_: CCSD(T)/CBS+CV+fT+pQ equilibrium rotational constants augmented by vibrational corrections (given in brackets) at the fc-CCSD(T)/cc-pVTZ level.

b Δ_*J*_, Δ_*K*_, Δ_*JK*_, δ_*J*_, δ_*K*_: ae-CCSD(T)/cc-pCVQZ level.

c A-reduced Watson Hamiltonian (*I*^*r*^ representation).

d χ_*aa*_, χ_*bb*_, χ_*ab*_: ae-CCSD(T)/cc-pCVQZ level; traceless symmetric tensor.

e μ_*a*_, μ_*b*_: fc-CCSD(T)/aug-cc-pVTZ level.

**Figure 3:**
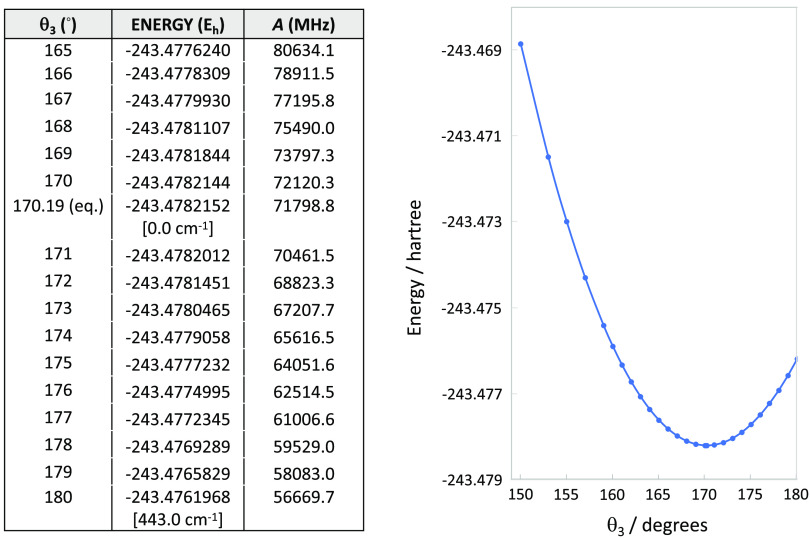
Minimum energy profile along the θ_3_ coordinate (right) at the fc-CCSD(T)/cc-pVTZ level. A selection of the energy values together with corresponding values of the *A* rotational constant is reported in the table (left panel: the θ_3_ value of 170.19° corresponds to the optimized geometry). Numbers in brackets provide the relative energy difference (in cm^–1^) between the equilibrium structure and the linear configuration of the NCO moiety.

In Figure 3, the PES profile at the fc-CCSD(T)/cc-pVTZ level along the θ_3_ coordinate is shown together with the corresponding variation of the *A* rotational constant. The points along the curve of Figure 3 have been obtained by fixing the θ_3_ value while optimizing all of the other structural parameters. It is noted that varying θ_3_ by 1° leads to a change in the *A* rotational constant greater than 1000 MHz (from ∼1720 MHz when moving from θ_3_ = 165° to θ_3_ = 166°, to ∼1410 MHz when moving from θ_3_ = 179° to θ_3_ = 180°). The results of Figure 3 indeed demonstrate how much such a rotational constant is sensitive to the θ_3_ coordinate.

### IR Spectrum

As explained in Section 2, the calculation of harmonic and anharmonic force fields also allows the prediction of the fundamental vibrational frequencies of HONCO, with the results collected in Table 3. The same approach was employed, for example, in the spectroscopic characterization of different isomers of cyanomethanimine.^65^ In that spectroscopic study, the availability of experimental values allowed for pointing out that *a posteriori* correction of harmonic frequencies at the ae-CCSD(T)/cc-pCVQZ level with anharmonic corrections at the fc-CCSD(T)/cc-pVTZ level leads to predictions with an average accuracy of ∼1%. However, it has to be noted that, in the present case, the anharmonic frequency correction for the lowest vibrational mode (ν_9_) seems to be suspiciously large (even if negative and in line with what is found in ref (8)), and the same applies to the anharmonic intensity correction for the ν_3_ mode. Nevertheless, the harmonic IR intensities (at the ae-CCSD(T)/cc-pCVQZ level) are expected to provide a good qualitative prediction for the band strength. While the ν_9_ fundamental has not been found significantly involved in any resonance, ν_3_ is affected by a Fermi resonance with the ν_4_+ν_7_ combination band, which is responsible for the intensity transfer leading to the large correction noted above.

**Table 3:** Harmonic Vibrational Frequencies ω, Anharmonic Corrections to Frequencies Δν, and Hybrid Fundamental Frequencies ν Are Reported in cm^–1^. Harmonic Infrared Intensities *I*_IR_ and Anharmonic Corrections to Infrared Intensities Δ*I*_IR_ Are Given in km mol^–1^

mode	irreducible representation	assignment	ae-CC/CQZ ω	fc-CC/VTZ Δν	hybrid anharmonic ν	ae-CC/CQZ harmonic *I*_IR_	fc-CC/VTZ anharmonic Δ*I*_IR_
ν_9_	*a*″	HONC out-of-plane torsion	210.9	–33.5	177.3	127.0	–20.9
ν_8_	*a*″	ONCO out-of-plane torsion	525.4	–0.3	525.1	9.7	0.8
ν_7_	*a*′	NOC bending	229.1	–8.5	220.6	8.8	0.7
ν_6_	*a*′	NCO bending	700.2	–4.7	695.5	18.7	–2.6
ν_5_	*a*′	NO stretching	894.4	–24.9	869.5	54.0	–5.1
ν_4_	*a*′	NO stretching + HON bending	1281.6	–36.6	1245.0	55.5	–9.3
ν_3_	*a*′	HON bending	1499.8	–51.8	1448.0	50.1	–52.8
ν_2_	*a*′	CN stretching + CO stretching	2266.0	–44.4	2221.6	664.0	–106.4
ν_1_	*a*′	OH stretching	3850.1	–190.2	3659.9	100.0	–22.4

**Figure 4:**
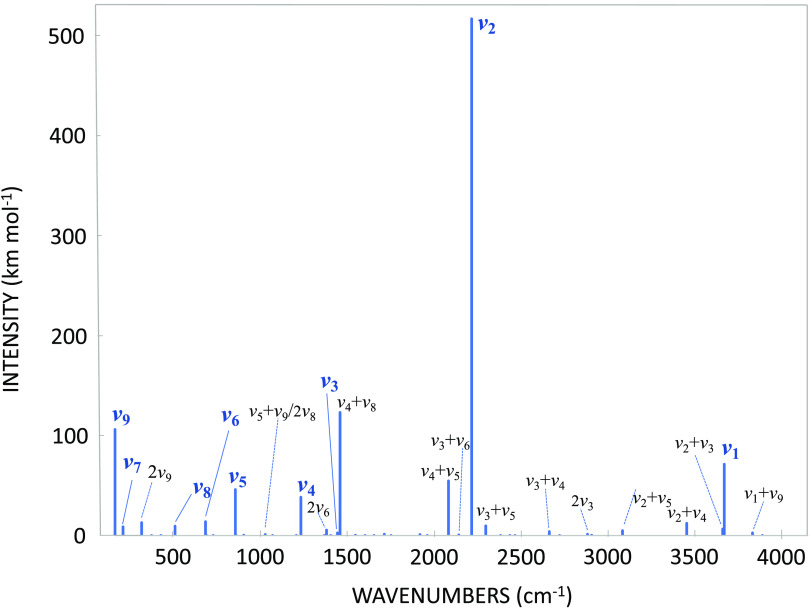
Simulation of a portion of the IR spectrum (100–4100 cm^–1^), up to two quanta, as obtained from VPT2 calculations on top of the fc-CCSD(T)/cc-pVTZ anharmonic force field. Band assignment is reported only for those features with an intensity greater than 1 km mol^–1^.

To provide an overview of the resulting IR spectrum, its simulation based on the anharmonic force field calculation carried out at the fc-CCSD(T)/cc-pVTZ level (for both line position and intensity), considering up to two quanta, is shown in Figure 4. It is noted that the IR spectrum up to about 2500 cm^–1^ is very rich, even if most of the features are extremely weak (i.e., having an intensity lower than 1 km mol^–1^). Above 4000 cm^–1^ (not reported in Figure 4), the only relevant band is the first overtone of the *v*_1_ band. The complete list of the vibrational transitions up to 4000 cm^–1^ is provided in the Supporting Information.

For comparison purposes, the IR spectrum of HNCO is considered. In ref (66), its six-dimensional PES of its X^1^A′ ground electronic state was evaluated at the ae-CCSD(T)/cc-pCVQZ level of theory. Variational calculations performed using such a PES led to the determination of the fundamental vibrational transitions with relative accuracy ranging between 0.2 and 0.5%. In view of the limited impact expected for the approximation of computing anharmonicity at a lower level of theory, the investigation of ref (66) tends to support the claimed accuracy of nearly 1% for our HONCO fundamental bands. Moreover, the study of ref (66) together with the harmonic force field of HNCO computed at the ae-CCSD(T)/cc-pCVQZ level allow us to discuss the differences in the vibrational features with respect to HONCO. First of all, it is noted that the lowest in-plane bending vibrational mode of HNCO lies at 574.4 cm^–1^ when incorporating anharmonicity^66^ (570.0 cm^–1^ at the harmonic level, the experimental gas-phase value being 577.4 cm^–1^ from ref (67)). This is mainly described by the NCO bending mode, which lies at higher energy in HONCO, i.e., at 700.2 cm^–1^ at the harmonic level (695.5 cm^–1^ from the hybrid model). Furthermore, the barrier to linearity along the NCO bending is larger in HONCO (443 cm^–1^ at the fc-CCSD(T)/cc-pVTZ level; see Figure 3) compared to HNCO (336 cm^–1^ at the ae-CCSD(T)/cc-pCVQZ level; see ref (66)). Thus, the effects of quasi-linearity are more pronounced in the case of HNCO, whereas HONCO is confirmed to behave as a bent molecule with respect to the NCO vibrational bending mode.

### Electronic Excited States and Photochemistry

**Figure 5:**
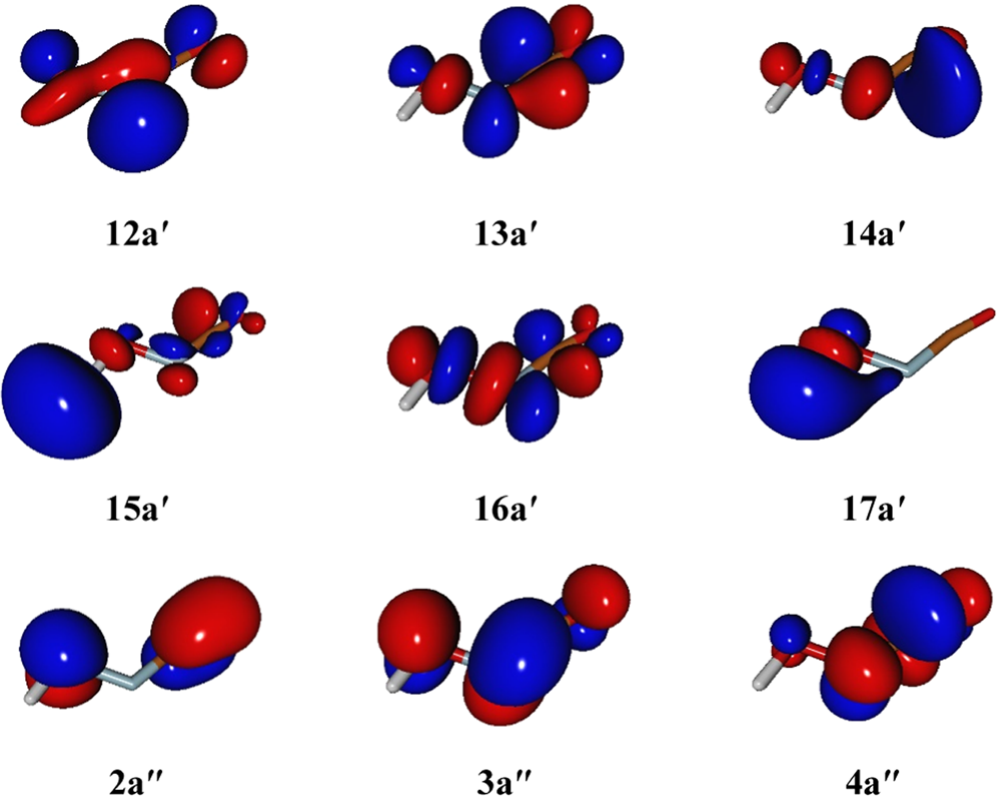
Outermost molecular orbitals at the ground-state equilibrium geometry of HONCO from SA-CASSCF/aug-cc-pVQZ calculations. The isodensity surfaces are traced at 0.05.

In Table 4, we provide the vertical excitation energies (*T*_v_) from the ground to the low-lying electronic states of HONCO calculated at the MRCI/aug-cc-pVQZ level. In these computations, as mentioned in Section 2, the triplet and singlet states were evaluated separately, and the molecular geometry was kept fixed at the CCSD(T)/CBS+CV+fT+pQ ground-state equilibrium structure (Table 1). The *T*_v_ value for the lowest triplet state (1 ^3^A″) has been computed at the fc-CCSD(T)/cc-pVQZ level. The MRCI transition energies for the higher triplet states were calculated by adding the appropriate energy differences to this reference value (3.73 eV). All of these transitions fall into the near-UV spectral region, with the lowest being to the 1 ^3^A″ state, at 3.73 eV. This particular transition results from the promotion of one electron from the 3a″ orbital to the 15a′ antibonding orbital (Figure 5). Strictly speaking, vertical transitions from the X ^1^A′ state to triplet states are not allowed by spin conservation. Consequently, these are expected to exhibit low intensity unless significant spin–orbit coupling, arising from the charge of the heavier atoms, weakens the Δ*S* = 0 selection rule enough to make them observable. Nevertheless, the T_0_ − S_0_ transition may be observed by phosphorescence (a priori in the UV domain).

**Table 4:** Vertical Excitation Energies (*T*_v_, eV) at the MRCI/aug-cc-pVQZ Level (MRCI+Q Values in Parentheses), Calculated at the CCSD(T)/CBS+CV+fT+pQ Optimized Geometry of S_0_ (See Table 1)^a^

	*T*_v_	*R*_e_	dominant electronic configuration
X ^1^A′ (S_0_)	0.00^b^	1.77	(2a″)^2^(12a′)^2^(3a″)^2^
1 ^3^A″ (T_1_)	3.73		(2a″)^2^(12a′)^2^(3a″)^1^(15a′)^1^
1 ^1^A″ (S_1_)	4.18 (4.12)	0.36	(2a″)^2^(12a′)^2^(3a″)^1^(13a′)^1^
1 ^3^A′ (T_2_)	5.55 (5.45)		(2a″)^2^(12a′)^1^(3a″)^2^(15a′)^1^
2 ^3^A″ (T_3_)	6.31 (6.43)		(2a″)^2^(12a′)^2^(3a″)^1^(17a′)^1^
2 ^3^A′ (T_4_)	6.56 (6.53)		(2a″)^2^(12a′)^2^(3a″)^1^(4a″)^1^
2 ^1^A″ (S_2_)	6.65 (6.68)	0.25	(2a″)^2^(12a′)^2^(3a″)^1^(14a′)^1^
2 ^1^A′ (S_3_)	6.66 (6.41)	1.62	(2a″)^2^(12a′)^1^(3a″)^2^(13a′)^1^
3 ^3^A″ (T_4_)	7.00 (7.05)		(2a″)^2^(12a′)^2^(3a″)^1^(14a′)^1^

a Transition and Dipole Moments (*R*_e_, D) at the SA-CASSCF/aug-cc-pVQZ level are given for the singlet states.

b Used as reference.

According to the symmetry selection rules for electronic transitions of planar polyatomic molecules, all of the transitions from the ground state to excited singlet states are possible. In HONCO, the strongest one is to the 2 ^1^A′ state at 6.66 eV, for which the electronic transition moment is calculated to be 1.62 D, and the second strongest one is to the 1 ^1^A″ state at 4.18 eV, with a transition moment of 0.36 D. All of the other transitions are weaker. The vertical transition to the 2 ^1^A′ state results from a one-electron excitation from the bonding 12a′ to the antibonding 13a′ orbital, while the transition to the 1 ^1^A″ state consists of the excitation of one electron from the 3a″ to the 13a′ orbital (see Figure 5). The lifetime for the S_0_ ← S_1_ transition can be evaluated according to the formula
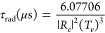
7where *R_e_* is the transition moment (in D) between the two states, here computed at the SA-CASSCF/aug-cc-pVQZ level, and *T*_v_ is the corresponding vertical excitation energy (in eV), evaluated at the MRCI/aug-cc-pVQZ level. This equation can be immediately deduced from the expression of Einstein coefficients for induced and spontaneous emission (*B*_*nm*_ and *A*_*nm*_, respectively) and from the relation τ = 1/∑_*m*_*A*_*nm*_. From eq 7, for the S_0_ ← S_1_ transition, a rather short radiative lifetime of 7.84 × 10^–3^ μs is computed.

**Figure 6:**
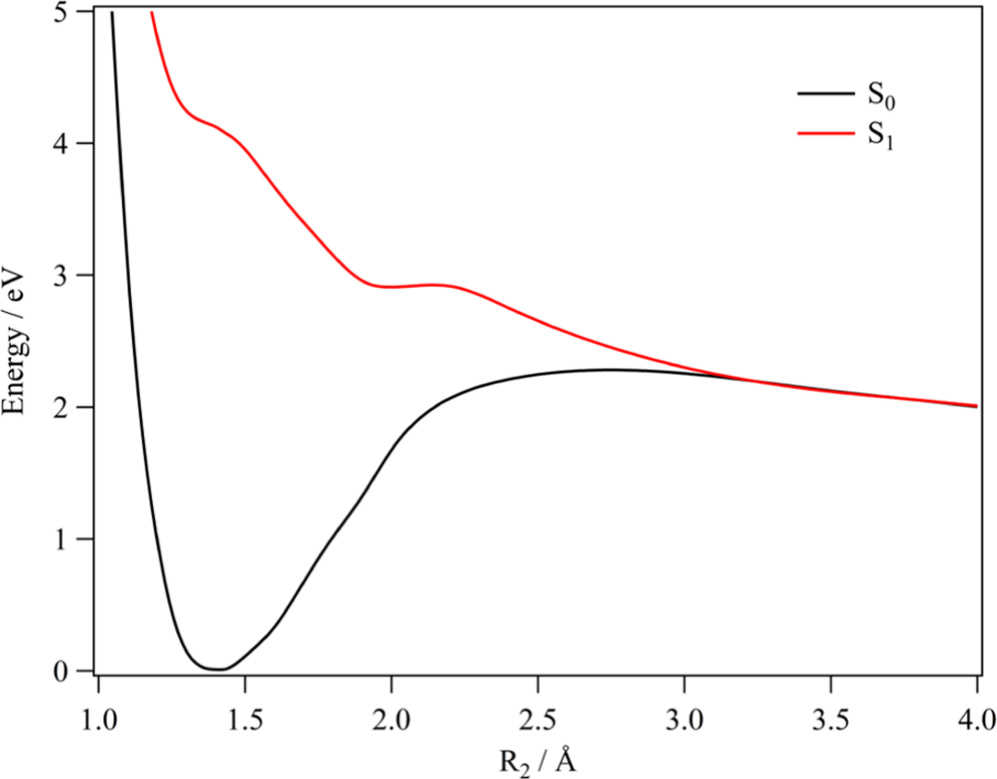
One-dimensional cut along the R_2_ distance (NO bond length) of the PESs of the S_0_ (X^1^A′) and S_1_ (1^1^A″) electronic states of HONCO, calculated at the MRCI/aug-cc-pVQZ level.

Figure 6 shows the computed one-dimensional cuts of the PESs for the S_0_ (X ^1^A′) and S_1_ (1^1^A″) states of HONCO along the NO distance (R_2_; Figure 1), as obtained at the MRCI/aug-cc-pVQZ level of theory. The other internal coordinates were kept fixed at their equilibrium CCSD(T)/CBS+CV+fT+pQ values for the electronic ground state (Table 1). From these cuts, the photodissociation dynamics of the HONCO molecule upon absorption of a photon of 4.2 eV energy can be qualitatively discussed. Indeed, upon absorption of a photon of sufficient energy, the system may either undergo an intramolecular rearrangement to a more stable geometry in the S_1_ excited state outside the Franck_Condon region or rather following the dissociative path illustrated in Figure 6, where the molecule remains planar during the process while undergoing a homolytic fragmentation into OH and NCO. Among the possible fragmentations, this process would be the most thermodynamically favorable because of the dissociative nature of the S_1_ state along this coordinate and the rather low bond enthalpy of the ON bond (∼55 kcal mol^–1^ on average) compared to the HO, N=C, and C=O bonds (all above 100 kcal mol^–1^). From Figure 6, we can infer a dissociation energy of around 2 eV (46 kcal mol^–1^).

## Discussion

Milligan et al.^20^ suggested a HONCO quasi-linear chain among the 1:1 NH:CO_2_ possible compounds originated from the photolysis of HN_3_ in solid CO_2_. The comparison of the fundamental frequencies from the hybrid approach with the features reported by Milligan et al.^20^ does not lead to any positive match. For most of them, there is no correspondence at all. Only for one feature (1213 cm^–1^),^20^ a similar value (ν_4_ at 1245 cm^–1^) is found in Table 3. In addition, the investigation of the S_0_ and S_1_ states of HONCO shows that this molecule is unstable upon UV–vis irradiation. Thus, this may explain why it was not observed in the early experiments of Milligan et al.,^20^ although being the most stable isomer. Indeed, these authors irradiated HN_3_ trapped in a CO_2_ cold matrix to form HNCO_2_ species. Such radiation would have destroyed the HONCO isomer, and solely the second most stable one, HNCO_2_ (compound II of ref (8)), is detected. In other words, the photolysis of HN_3_ in a CO_2_ cold matrix is not an efficient way for the production of such molecules.

As mentioned in the Introduction, HONCO can be formed in the complex chemistry of HCN/HNC in oxygen-rich gaseous mixtures or via the CH + NO_2_, NH + CO_2_, N + HOCO, and HCO + NO reactions,^3−7^ which are relevant for combustion and atmospheric chemistry. Concerning the latter, the UV–vis light present in the Earth’s atmosphere leads to its photodissociation at least via the S_1_ state. Thus, if present in this medium, HONCO is expected to lie in its electronic ground state. On the other hand, its photodissociation should participate in the modification of the atmospheric NCO radical budget and, more importantly, that of the OH radical. Moving to the ISM, the NCO radical is the smallest species containing the backbone of the peptide bond, and, therefore, the observation of small molecules bearing such a moiety can provide important clues on the possible formation of amino acids in space. NCO is predicted to be abundant in dark clouds and is considered the main precursor of HNCO.^18,68^ However, the chemistry of NCO at the low temperatures of interstellar clouds has been scarcely investigated so far. In this respect, in parallel to experimental spectroscopic characterizations of HONCO, gas-phase reaction pathways leading to its formation deserve to be investigated.

## Conclusions

Using the first-principles methodology, we have characterized the most stable isomer of the [H,C,N,O,O] family: for the quasi-linear chain HONCO molecule, accurate structural, rotational, vibrational, and electronic properties have been determined. Despite its quasi-linearity and a torsional mode lying at ∼177 cm^–1^, HONCO is a rather well-behaved semirigid molecule. Therefore, our state-of-the-art computational investigation allowed us to provide accurate predictions for the rotational and IR spectra, which lay the foundation for experimental characterization. The study has also been extended to the lowest electronic states, which permits one to provide the pattern of its singlet and triplet electronic states lying in the 0–7 eV energy domain to be determined. All of these data are expected to be of great help in the characterization of HONCO in atmospheric, astrophysical, and combustion media. In particular, we showed that HONCO exhibits dissociation upon absorption of UV–vis light, thus producing the NCO and OH radicals. This means that HONCO is expected to participate in their budget in the Earth’s atmosphere, thus confirming its importance as an intermediate species in atmospheric chemistry.

Finally, it is noteworthy that sulfur (singly and doubly)-substituted analogues of HONCO and their isomers (e.g., hypothiocyanous acid, HOSCN,^69,70^ and sulfenyl thiocyanate^71^) play an important role in biology and in biochemistry. Therefore, on one side, a possible role of the [H,C,N,O,O] family in this respect deserves to be investigated in the future, and on the other, we suggest to extend the present accurate methodologies to the characterization of the sulfur-substituted analogues with the aim of their identification in the laboratory and *in vivo*.

## Supporting Information Available

A list of the vibrational transitions up to two quanta in the 0–7150 cm^–1^ interval evaluated at the fc-CCSD(T)/cc-pVTZ level (PDF)

## Footnotes

a The symbol ^*x*^*M*_δ*Ka*,δ*Kc*_ is used to identify the transition type of an asymmetric rotor: *x* stands for the dipole moment component involved (*a* and, thus μ_a_, in the present case); *M = P, Q*, or *R* denotes variation of *J* (the rotational quantum number) in the transition, with Δ*J* = −1, 0, or +1, respectively (*R* with Δ*J* = +1 in the present case); δ*K*_*a*_ and δ*K*_*c*_ stand for the (signed) change of the *K*_*a*_ and *K*_*c*_ pseudo quantum numbers of the asymmetric rotor (0 and 1 in the present case).
