# Supplementary material for: Insights into the Molecular Structure and Spectroscopic Properties of HONCO: An Accurate Ab Initio Study
Source: J Phys Chem A. 2023 Nov 3;127(45):9502–12. doi: 10.1021/acs.jpca.3c05741 (PMC10658623; doi:10.1021/acs.jpca.3c05741)
Supplement: Supplementary file 1 — jp3c05741_si_001.pdf [file jp3c05741_si_001.pdf]

Insights into the molecular structure and spectroscopic properties of HONCO: An accurate *ab initio* study

Cristina Puzzarini,[a,*] Roberto Linguerri,[b,*].Majdi Hochlaf [b,*].

[a] Dipartimento di Chimica "Giacomo Ciamician", Università di Bologna, Via Selmi 2, 40126 Bologna, Italy.

[b] Université Gustave Eiffel, COSYS/IMSE, 5 Bd Descartes 77454, Champs sur Marne, France.

SUPPORTING INFORMATION

VIBRATIONAL TRANSITIONS (up to two quanta) from VPT2 ANALYSIS
Level of theory: fc-CCSD(T)/cc-pVTZ

MODES Upper level I	J	K	L	M	MODES Lower level NI	NJ	NK	NL	NM	Anharmonic Frequency (cm^{-1})	Anharm Intensity (km/mol)	Harmonic Transition (cm^{-1})
7	0	0	0	0	1	0	0	0	0	171.420	106.243920	204.971321
8	0	0	0	0	1	0	0	0	0	217.687	8.954212	226.197300
7	0	0	0	0	2	0	0	0	0	323.737	13.140118	409.942643
8	7	0	0	0	1	1	0	0	0	381.133	0.248382	431.168622
8	0	0	0	0	2	0	0	0	0	434.458	0.075309	452.394601
9	0	0	0	0	1	0	0	0	0	514.000	9.434540	514.300861
10	0	0	0	0	1	0	0	0	0	689.907	14.015508	694.557822
9	7	0	0	0	1	1	0	0	0	693.240	0.292613	719.272182
9	8	0	0	0	1	1	0	0	0	732.533	0.000442	740.498161
11	0	0	0	0	1	0	0	0	0	858.187	46.095370	883.099006
10	7	0	0	0	1	1	0	0	0	862.937	0.041579	899.529144
10	8	0	0	0	1	1	0	0	0	907.178	0.710448	920.755123
11	7	0	0	0	1	1	0	0	0	1029.874	0.049704	1088.070328
9	0	0	0	0	2	0	0	0	0	1029.951	1.169091	1028.601721
11	8	0	0	0	1	1	0	0	0	1071.915	0.233953	1109.296307
10	9	0	0	0	1	1	0	0	0	1207.392	0.017754	1208.858683
12	0	0	0	0	1	0	0	0	0	1232.944	38.412499	1269.536340
11	9	0	0	0	1	1	0	0	0	1367.783	0.003447	1397.399867
10	0	0	0	0	2	0	0	0	0	1380.355	5.696109	1389.115645
12	7	0	0	0	1	1	0	0	0	1403.252	0.005167	1474.507662
13	0	0	0	0	1	0	0	0	0	1441.411	2.805837	1493.222727
12	8	0	0	0	1	1	0	0	0	1456.939	123.087638	1495.733641
11	10	0	0	0	1	1	0	0	0	1545.398	0.517918	1577.656829
13	7	0	0	0	1	1	0	0	0	1600.395	0.231850	1698.194048
13	8	0	0	0	1	1	0	0	0	1650.039	0.129701	1719.420027
11	0	0	0	0	2	0	0	0	0	1709.178	1.602406	1766.198013
12	9	0	0	0	1	1	0	0	0	1748.311	0.108804	1783.837201
12	10	0	0	0	1	1	0	0	0	1913.566	1.263395	1964.094163
13	9	0	0	0	1	1	0	0	0	1955.593	0.048277	2007.523587
12	11	0	0	0	1	1	0	0	0	2075.726	54.944385	2152.635347
13	10	0	0	0	1	1	0	0	0	2134.380	1.017628	2187.780549
14	0	0	0	0	1	0	0	0	0	2208.946	516.742446	2253.363298
13	11	0	0	0	1	1	0	0	0	2289.699	9.970595	2376.321733
14	7	0	0	0	1	1	0	0	0	2373.223	0.001765	2458.334620
14	8	0	0	0	1	1	0	0	0	2426.270	0.025260	2479.560599
12	0	0	0	0	2	0	0	0	0	2455.207	0.206801	2539.072681
13	12	0	0	0	1	1	0	0	0	2652.151	4.072679	2762.759067
14	9	0	0	0	1	1	0	0	0	2709.791	0.027275	2767.664159
13	0	0	0	0	2	0	0	0	0	2870.659	1.611941	2986.445454
14	10	0	0	0	1	1	0	0	0	2893.132	0.482351	2947.921121
14	11	0	0	0	1	1	0	0	0	3070.050	5.070597	3136.462305
14	12	0	0	0	1	1	0	0	0	3436.615	12.325544	3522.899639
14	13	0	0	0	1	1	0	0	0	3641.806	6.635504	3746.586025
15	0	0	0	0	1	0	0	0	0	3651.400	71.433032	3841.601918
15	7	0	0	0	1	1	0	0	0	3812.904	2.774011	4046.573240
15	8	0	0	0	1	1	0	0	0	3868.519	0.111074	4067.799219
15	9	0	0	0	1	1	0	0	0	4164.766	0.019596	4355.902779
15	10	0	0	0	1	1	0	0	0	4340.893	0.020595	4536.159741
14	0	0	0	0	2	0	0	0	0	4392.388	0.823361	4506.726596
15	11	0	0	0	1	1	0	0	0	4507.339	0.079581	4724.700925
15	12	0	0	0	1	1	0	0	0	4878.129	0.163580	5111.138259
15	13	0	0	0	1	1	0	0	0	5072.424	0.504530	5334.824645
15	0	0	0	0	2	0	0	0	0	7133.060	6.810697	7683.203837

Legend

7 = ν_9 8 = ν_7 9 = ν_8 10= ν_6
11= ν_5 12= ν_4 13= ν_3 14= ν_2
15= ν_1
